# Supplementary figures and images for: Structure of Aedes aegypti procarboxypeptidase B1 and its binding with Dengue virus for controlling infection
Source: Life Sci Alliance. 2021 Nov 8;5(1):e202101211. doi: 10.26508/lsa.202101211 (PMC8605224; doi:10.26508/lsa.202101211)

## Slide 1
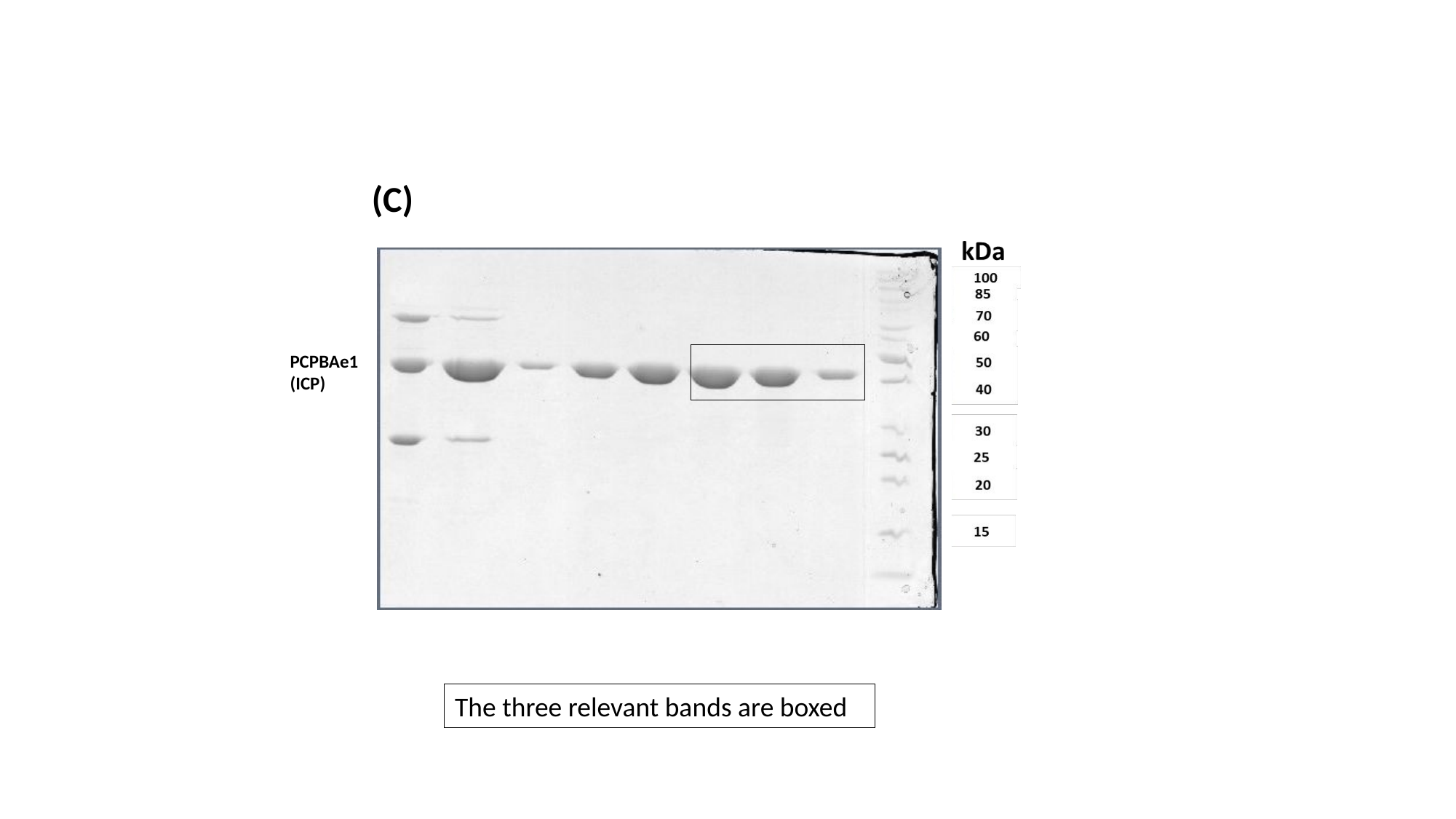

(C)
PCPBAe1
(ICP)
The three relevant bands are boxed

Supplement: Supplementary file 1 [file LSA-2021-01211_SdataFS1.1.pptx]

## Slide 1
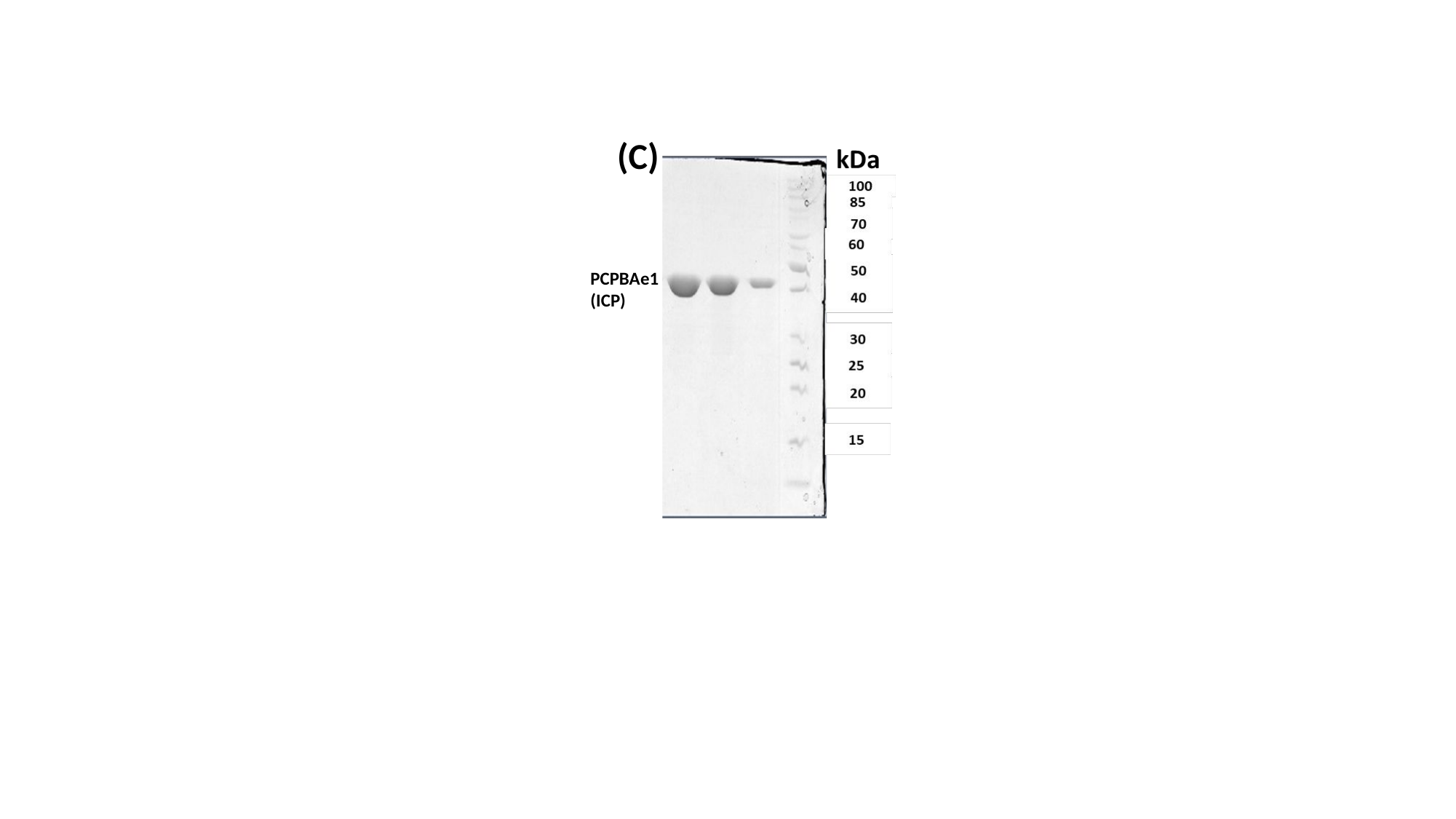

(C)
PCPBAe1
(ICP)

Supplement: Supplementary file 2 [file LSA-2021-01211_SdataFS1.2.pptx]
