# Supplementary material for: Structure of Aedes aegypti procarboxypeptidase B1 and its binding with Dengue virus for controlling infection
Source: Life Sci Alliance. 2021 Nov 8;5(1):e202101211. doi: 10.26508/lsa.202101211 (PMC8605224; doi:10.26508/lsa.202101211)
Supplement: Supplementary file 3 [file LSA-2021-01211_TableS1.docx]

Supplementary table S1. Structural comparison of different PCPs with PCPBAe1

| Species/Name | PDB ID | RMSD (Å) | % Sequence identity | No. of Cα |
| --- | --- | --- | --- | --- |
| PCPAHa | 1JQG | 1.4 | 39 | 394 |
| PCPBh | IKWM | 1.6 | 36 | 394 |
| PCPB | 1NSA | 1.5 | 34 | 394 |

PCPAHa-procarboxypeptidase B from Helicoverpa armigera, PCPBh-procarboxypeptidase B from human, PCPB-procarboxypeptidase B from porcine. Analysis was done with DALI server.
