# Supplementary material for: Structure of Aedes aegypti procarboxypeptidase B1 and its binding with Dengue virus for controlling infection
Source: Life Sci Alliance. 2021 Nov 8;5(1):e202101211. doi: 10.26508/lsa.202101211 (PMC8605224; doi:10.26508/lsa.202101211)
Supplement: Supplementary file 4 [file LSA-2021-01211_TableS2.docx]

Supplementary table S2. Crystallographic table and refinement statistics for PCPBAe1 structure

|  | PCPBAe1 |
| --- | --- |
| **Data collection** |  |
| Unit cell (Å) | a=139.79; b=74.44; c=83.11 |
| Space group | C2_1_ |
| Resolution range (Å) | 50 – 2.08 (2.12-2.08) * |
| Wavelength (Å) | 1.5418 |
| Total reflections | 220441 |
| Unique reflections | 44087 |
| Completeness (%) | 98.53% (95.54%) |
| Overall (I/σI) | 27.74 (5.25) * |
| Redundancy | 5.0 (4.3) * |
| R_Sym_^a^ | 0.067(0.159) |
| **Refinement and quality** |  |
| Resolution range (Å) | 34.85 – 2.08 |
| R_work_^b^ | 0.167 |
| R_free_^c^ | 0.196 |
| RMSD bond length (Å) | 0.003 |
| RMSD bond angles (^o^) | 0.59 |
| **Average B factors** |  |
| Main chain atoms (Å^2^) | 17.90 |
| Ligands | 24.52 |
| Water | 27.13 |
| **Ramachandran plot ^d^** |  |
| Favored region (%) | 98.27% |
| Allowed regions (%) | 1.73% |
| Disallowed region (%) | 0.00% |

* Values in parentheses correspond to the highest resolution shell

aRmerge = ∑∑ | I (k)—<I>| / ∑ I (k) where I (k) and <I> represent the diffraction intensity values of the individual measurements and the corresponding mean values. The summation is over all unique measurements.

bRwork = ∑ ||Fobs|—k|Fcalc|| / |Fobs| where Fobs and Fcalc are the observed and calculated structure factors, respectively.

cRfree is the sum extended over a subset of reflections (10%) excluded from all stages of the refinement.

dAs calculated using MolProbity.
