# Supplementary material for: Structure of Aedes aegypti procarboxypeptidase B1 and its binding with Dengue virus for controlling infection
Source: Life Sci Alliance. 2021 Nov 8;5(1):e202101211. doi: 10.26508/lsa.202101211 (PMC8605224; doi:10.26508/lsa.202101211)
Supplement: Supplementary file 5 [file LSA-2021-01211_TableS3.docx]

Supplementary table S3. Site-directed mutagenesis of PCPBAe1 and oligonucleotides

| Peptide | Mutation | Oligonucleotide (5’-3’) |
| --- | --- | --- |
| Asp^251^Glu |  | GCGGCGGGTGGTAGCGAAGATTGGGCGTTCGCG  CGCGAACGCCCAATCTTCGCTACCACCCGCCGC |
| Ser^239^Gly |  | TACACCGTGGGTAGCGGTACCAACGTTCTGTAT  ATACAGAACGTTGGTACCGCTACCCACGGTGTA |
| Tyr^4A^-Glu^21A^ | Asp^18A^Glu^19A^ del | GTTCCGGAAAGCCCGGCGGAAATCCTGTAT  ATACAGGATTTCCGCCGGGCTTTCCGGAAC |
| Ala^81A^-Glu^20^ | Asp^5^Ala, Arg^6^Ala,  Asp^7^Ala | GGCATGAGCATGCTGGCGGCGGCGGTGAGCACCAGCTAC  GTAGCTGGTGCTCACCGCCGCCGCCAGCATGCTCATGCC |
| Gly^162^-Arg^172^ | Glu^168^Ala, Glu^170^Ala, Arg^172^Ala | GAAACCGCGTTTAGCGCGCCGGCGACCGCGGCGGTGCGTGATGCG  CGCATCACGCACCGCCGCGGTCGCCGGCGCGCTAAACGCGGTTTC |
| Val^300^-Phe^305^ del | Val^300^-Phe^305^ del | GCGATGGCGCTGAAATAAGTTGCGCAAATGTTT  AAACATTTGCGCAACTTATTTCAGCGCCATCGC |
| ProCP | ProCP | AGCATGCTGGACTAGCGTGATGTGAGC  GCTCACATCACGCTAGTCCAGCATGCT |
