# Supplementary material for: Structure of Aedes aegypti procarboxypeptidase B1 and its binding with Dengue virus for controlling infection
Source: Life Sci Alliance. 2021 Nov 8;5(1):e202101211. doi: 10.26508/lsa.202101211 (PMC8605224; doi:10.26508/lsa.202101211)
Supplement: Supplementary file 6 [file LSA-2021-01211_TableS4.docx]

Supplementary table S4. Oligonucleotides for the generation of de-glycosylated DENV-2 mutants

| Primer Name | Oligonucleotide (5’-3’) |
| --- | --- |
| D2Y98P-F1-FP | CTGGTTTAGTGAACCGTCAGAGTAGTTAGTCTACGTGGAC |
| D2Y98P-F1-RP | CTCACAACGCAACCACTATCGGCCTGCACCATAACTCC |
| D2Y98P-F2-FP | TGGGAGTTATGGTGCAGGCCGATAGTGGTTGCGTTGTG |
| D2Y98P-F2-RP | ATTGCTGGAAGGTATCTCTTTGTTTTTCCTGCTCCTGG |
| D2Y98P-F3-FP | ACCCAGGAGCAGGAAAAACAAAGAGATACCTTCCAGCAATAGTCAGAGAAG |
| D2Y98P-F3-RP | TTTGAAGACGCACCAGATTCCAACCATATGTTGACATGG |
| D2Y98P-F4-FP | CCCATGTCAACATATGGTTGGAATCTGGTGCGTCTTCAAAG |
| D2Y98P-F4-RP | TGGAGATGCCATGCCGACCCAGAACCTGTTGATTCAAC |
| Vector (CMV, HDV ribozyme and SV40 PA) FP | CTGTTGAATCAACAGGTTCTGGGTCGGCATGGCATCTC |
| Vector (CMV, HDV ribozyme and SV40 PA) RP | GTCCACGTAGACTAACTACTCTGACGGTTCACTAAACCAGC |
| 67-69 NTT-QTV FP | CAAAGCTGACCCAAACAGTTACAGCATCTCGCTGCCCAAC |
| 67-69 NTT-QTV RP | CGAGATGCTGTAACTGTTTGGGTCAGCTTTGCCTCTATAC |
| 153 N-Q FP | TGCAGTCGGACAAGACACAGGAAAAC |
| 153 N-Q RP | TCCGACTGCATTCTCTTCCCCTG |
